# A wideband and compact Quasi-Yagi antenna based on spoof surface plasmon polaritons

**DOI:** 10.1038/s41598-023-37419-z

**Published:** 2023-07-08

**Authors:** Farshad Arghandeh, Bijan Abbasi-Arand, Maryam Hesari-Shermeh

**Affiliations:** Department of Electrical and Computer Engineering, Tarbiat Modares University, Tehran, 14115-194 Iran

**Keywords:** Electrical and electronic engineering, Engineering

## Abstract

In this paper, a novel wideband end-fire antenna, based on a spoof surface plasmon polaritons (SSPP) transmission line, is proposed. Periodically modulated corrugated metal strips are used as a transmission line for quasi-TEM conversion in the microstrip line to the state of SSPP and the best impedance matching. Due to the strong confinement of the field in the SSPP waveguide and its high transmission performance, it has been used as a transmission line. The antenna consists of SSPP waveguides for the transmission line, a metal plate on the ground as the reflector of the antenna, a metal strip director, and two half-rings to realize the radiation, reaching a wide bandwidth in the range of 4.1 to 8.1 GHz. The simulation results show that this antenna achieves a gain of 6.5 dBi, a bandwidth of 65%, and an efficiency of 97% across a wide operating frequency band, from 4.1 to 8.1 GHz. The proposed end-fire antenna has been fabricated, and the measured results agree well with the simulated results. The end-fire antenna implemented on a dielectric layer also has the advantages of high efficiency, good directivity, high gain, a wide bandwidth, easy fabrication, and a compact size.

## Introduction

With recent advancements in wireless communication technology, the demand for low-profile wideband antennas is increasing. Yagi-Yoda antennas can be used for long-distance wireless communications due to their advantages, such as high gains, high bandwidths, low profiles, end-fire radiation patterns, and ease of fabrication and integration with other microwave circuits^1,2^. The Yagi-Uda antenna has a driving element, a reflector, and one or more directors.

In the literature, different designs have been reported to increase the bandwidths of planar-printed quasi-Yagi antennas. For example, a quasi-Yagi antenna based on a microstrip transmission structure was presented; and while the measured bandwidth was 48%, the structure of that antenna was complex^3^. Moreover, the driver and director was reported that printed dipoles positioned on one side of a substrate with a high dielectric permittivity^4^; while the reflector was a truncated ground plane positioned on the other side of the substrate. The feeding method was geometrically complex, and needed long transmission lines^4^. In addition, the use of a narrowband delay line in the construction of the wideband antenna restricts the antenna's bandwidth, and creates an unbalanced operating situation^5^. In one study of quasi-Yagi antenna, a wide bandwidth of 44% was obtained using a simple structure and feed^6^; however, the asymmetrical nature of the printed quasi-Yagi antenna made the radiation pattern bad^6^. Also, a quasi-Yagi antenna based on a microstrip-to-slit transmission structure was presented, and it reached a bandwidth of approximately 46%^7^; however, that antenna had a large ground, which increased the size of the antenna^7^.

Furthermore, a compact planar printed quasi -Yagi antenna with a modified ground plane, based on a new structure of microstrip-to-slit transmission line, was proposed^8^, which had an impedance bandwidth of 3.6 to 11.6 GHz, and a compact size^8^. Moreover, in another report of loop quasi -Yagi antenna, a loop Yagi-Uda antenna was used to reduce the antenna size by using the half-loop technique; and a measured bandwidth of 43.3% was obtained^9^. In addition, a comparison was made between two loop Yagi antennas—one with a defective ground structure (DGS), and the other without^10^. The antenna without the DGS had a better bandwidth, front-to-back ratio (FBR) gain and directivity, but it was 52% bulkier^10^. Therefore, it is challenging to design antennas with both high bandwidths and simple structures.

One way to increase the bandwidth is to use SSPP structures in the design of the antennas, where SPPs are the hybrid excitations produced when the SPs are coupled with electromagnetic (EM) waves^11^. The SPPs generate both charge oscillations in the metal and evanescent electromagnetic waves in the dielectric, and despite the fact that SPs and SPPs have been found at optical frequencies, they are closely related to surface waves at radio and microwave frequencies^12,13^. In 2004, for instance, Pendry et al. demonstrated a comparable surface plasmonic phenomenon known as SSPPs at microwave frequencies^14^.

SSPPs are therefore good candidates for high-density integrated circuits and components at millimeter and terahertz frequencies, due to their strong field confinements at the wavelength scale, as well as their groundless structures^15–17^. Recently, SSPPs have been widely used in optoelectronic technologies^18^, filters^19–21^, amplifiers^22^, antennas^23–30^, and antennas reconfigure^31–34^, due to their special properties. In one paper, the authors used a metamaterial structure to achieve end-fire radiation^26^; moreover, in another one, high-gain and wideband SSPPs with circular patch arrays were presented^27^.

In one of the recent reports of SSPP, proposed a method for designing common aperture antennas based on (SSPPs) excitations, which are similar to microwave to (SPPs) in the optical frequency region. In this paper, a common aperture antenna has been designed based on both odd and even SSPP modes^30^. Furthermore, a small aperture end-fire antenna based on odd-mode SSPPs was presente^11^; where SSPPs antennas were composed of dipole-unit cells, which reached bandwidths in the range of 4–8 GHz^11^. However, most of the structures presented in the literature are not suitable for practical applications, due to their high fabrication costs and complex structures. Therefore, designing an antenna with a small size and high bandwidth is challenging.

In this paper, an SSPP-based wideband antenna with high gains is proposed, where this SSPP waveguide is made up of a single ultra-thin, corrugated metal strip and a dielectric substrate layer. Here, the SSPP is used to transmit energy, while to realize the energy radiation, a Yagi loop has been added in the end-fire direction. In comparison to other transmission lines, this SSPP-fed antenna can simultaneously preserve the signal’s integrity, and it also incurs low conduction losses. To improve the directivity of the SSPP antenna, one director is also loaded at the end of the antenna. Our proposed antenna is simple, and has a better bandwidth, gain, and efficiency compared to other antennas presented in the literature^7,25,27^. It is shown, here, that the bandwidth, efficiency, and gain of our proposed antenna are approximately 65%, 97%, and 6.6 dBi, at the designed frequency of 6 GHz, respectively.

The rest of the paper is organized as follows: Section “Design implications” describes the geometry and specifications of the proposed antenna. Section “Fabrication and measurement”.presents the simulation and measurement results, to demonstrate the performance of the antenna; and finally, the paper is concluded in Section “Conclusion”.

## Design implications

**Figure 1:**
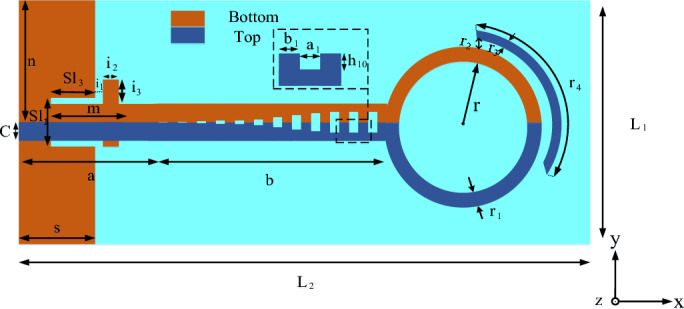
Schematic of our proposed antenna, based on SSPP.

The configuration of our proposed wideband end-fire antenna, based on SSPP, is shown in Fig. 1. This antenna is designed on a Rogers RO4350 substrate, with ${\varepsilon }_{r}=3.66$, $\mathrm{tan}\delta =0.0037$, and a thickness of t = 0.76 mm. The optimized antenna parameters are presented in Table 1.

**Table 1:** Optimum parameters of the SSPP-based antenna.

Parameter	Value (mm)	Parameter	Value (mm)	Parameter	Value (mm)
l1	40	a	22.5	r1	2.3
l2	85	b	31.2	r2	1.1
S	11	m	17.37	r3	1.3
Sl1	6	i1	0.5	r4	23.35
Sl3	5.92	i2	3.5		
N	17.8	i3	2.1		
C	2.2	r	10.5		

The designed antenna consists of five parts. A metal ground sheet is used on the bottom layer of the substrate, to improve the directionality, reduce the losses, and enhance the FBR of the antenna. Two slots created in front of the ground plane are then used as the antenna’s reflectors, which reflect the energy transmitted back, and this also improves the antenna’s radiation. Moreover, a pair of SSPP waveguides have been used as a transmission line to excite the end-fire antenna. This is due to its advantages, including high-field confinement, low losses, controllable dispersion, and slow waves. In addition, the tapered SSPP line provides superior impedance matching to open space, thereby suppressing any end-to-end reflections.

Different methods are used in the literature for matching the SSPP transmission line. One of these methods is using the characteristic impedance matching method^35^. In this paper, the gradient corrugated grooves structure is used to match the SSPP transmission line.

It is worth noting that the concept of an SSPP waveguide was used in reference^26^, but we have extended this concept to excite end-fire loop Yagi antennas. Thus, our proposed design increases the bandwidth and efficiency, by preserving the same dimensions while also having a simpler structure compared to^26^. To achieve impedance matching, corrugated grooves have been used between the input microstrip line and the SSPP waveguide, where the parameters of the corrugated metal strips are given in Table 2.

**Table 2:** The parameters of the metal strips with corrugated grooves.

Parameter	Value (mm)	Parameter	Value (mm)
h1	0.04	h6	0.68
h2	0.168	h7	0.808
h3	0.296	h8	0.936
h4	0.424	h9	1.064
h5	0.552	h10	1.2

As can be seen in Fig. 1, the SSPP units consist of metal strips with wavy grooves, where a1 and h10 represent the length and depth of the grooves, respectively. A metal strip director and two half-loops have been chosen to realize the end-fire radiation. As shown in Fig. 1, half of the driving loop element has been designed on the top layer (the blue loop) and the other half (the orange loop) has been designed on the bottom layer.

**Figure 2:**
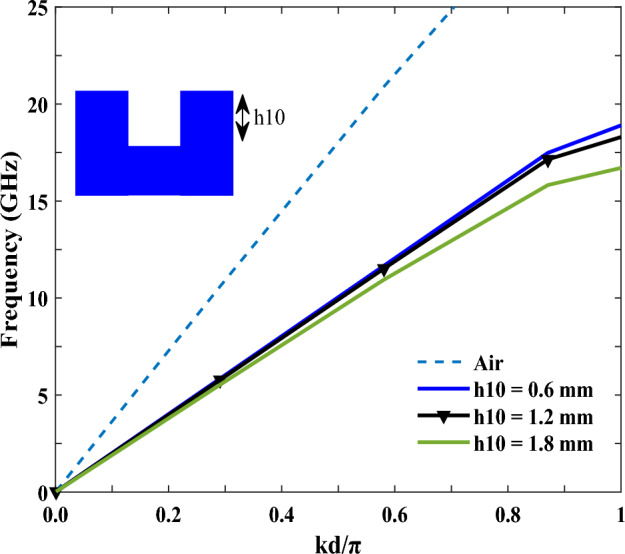
The curve of dispersion of the unit cell in the SSPP transmission line.

It is worth noting that the proposed antenna is based on the Hanson-Woodyard condition (HWC)^36^, meaning all of its parts contribute to the increase in radiation. According to the HWC for the end-fire radiation, the appropriate phase constant for the guided waves should be slightly larger than in the air. Yet, on the other hand, the dispersion curve needs to be under the airline, and also within a short distance. So, the dispersion curve of the unit cell has been simulated in CST software, as illustrated in Fig. 2 (see the inset for the unit cell). As can be seen in the figure, the dispersion diagram was below the airline; hence, the slow wave mode was the dominant mode, and the HWC for the proposed antenna has been fulfilled. As seen, the dispersion curve can be adjusted by varying the groove depth (h10). It is also worth noting that, with deeper grooves, a stronger field confinement is provided in the SSPPs’ transmission line, which leads to an improvement in the bandwidth, and a gain for the antenna. However, with a further increase of h10, the dispersion curve moves away from the airline, as shown in Fig. 2, which causes poorer phase matching, and free space for the radiation.

**Figure 3:**
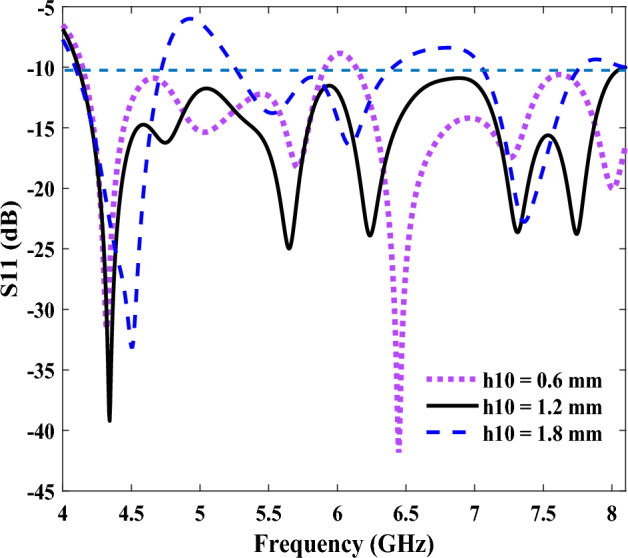
The reflection coefficients (S11) of the proposed antenna, for various groove depths.

Therefore, the effect of the groove depth on the impedance matching has been analyzed, to achieve an optimum value of h10 for wide bandwidths in the frequency range of 4–8 GHz. As shown in Fig. 3, the optimum value of h10 is 1.2 mm, in this frequency range; while the other dimensions of the structure have also been optimized, in a similar way, which are given for h10. For example, the optimized size of the ground plane is S = 11 mm; however, if the ground plane is smaller, the mode conversion to SSPP is not good, and as a result, it reduces both the bandwidth and the gain of the antenna. Moreover, if the ground plane is too large, it causes some unwanted radiation; and hence, it reduces the bandwidth and gains. Therefore, there is an optimal size for both the ground plane and the other parameters mentioned, in Tables 1 and 2. The results are illustrated in the frequency range of 4.1 to 8.1 GHz as the proposed antenna has a good impedance matching (s11 < − 10 dB) at this range.

**Figure 4:**
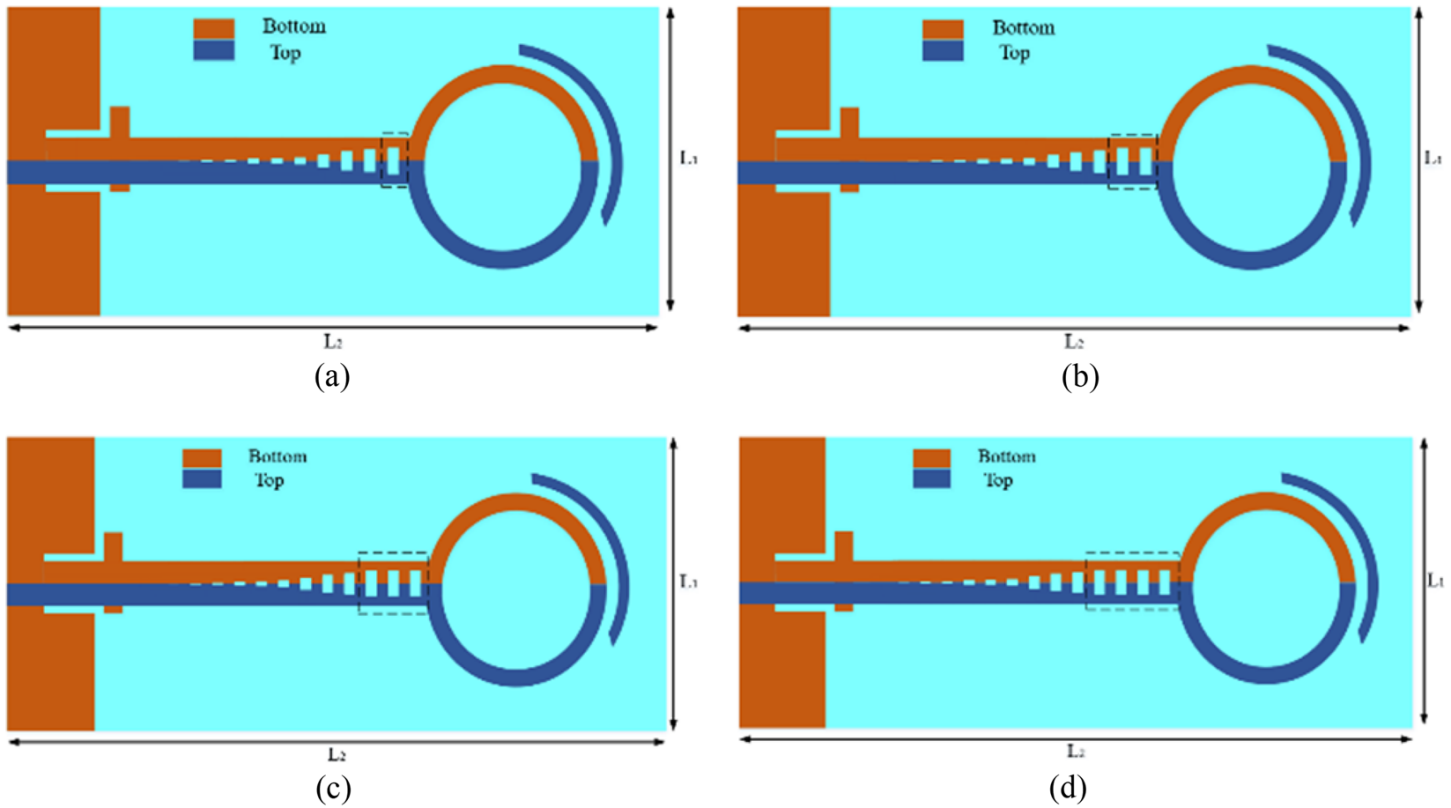
Schematics of an SSPP-based antenna. (**a**) Antenna schematic with a single-unit cell (n = 1). (**b**) Antenna schematic with two-unit cells (n = 2). (**c**) Antenna schematic with three-unit cells (n = 3). (**d**) Antenna schematic with four-unit cells (n = 4).

**Figure 5:**
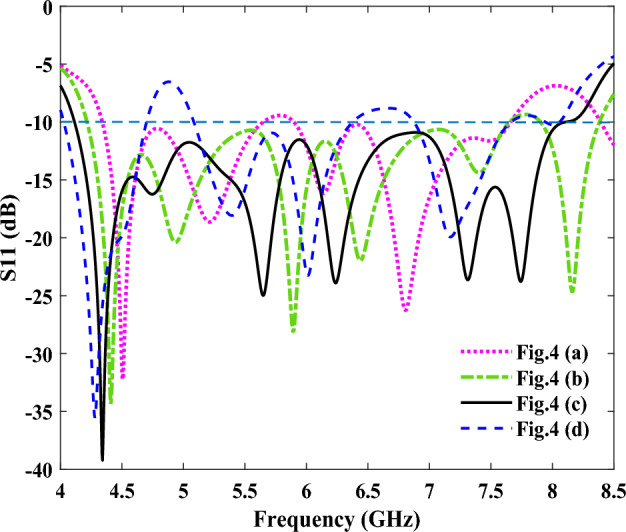
The simulated reflection coefficients of the four antennas shown in Fig. 4.

**Figure 6:**
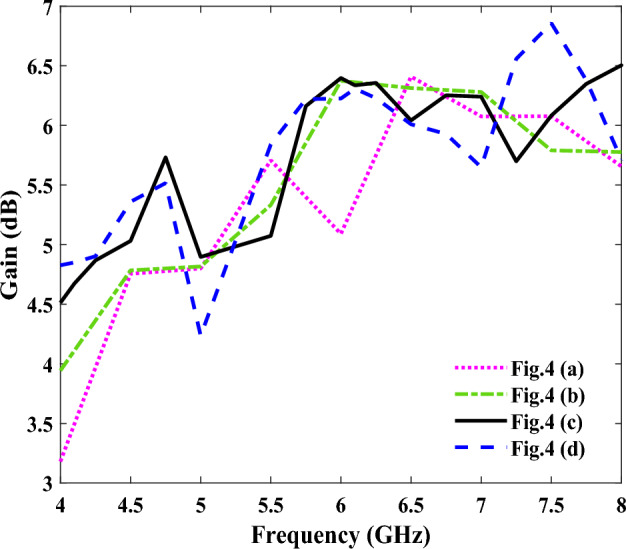
The simulated gains of the four antennas shown in Fig. 4.

The number of unit cells of the groove of the SPP transmission line have also been optimized, where a schematic of quasi-Yagi antennas with four different unit cell numbers is shown in Fig. 4. Figure 4a shows our initial design, with a single-unit cell SSPP and length l2 = 79.8 mm, while the reflection coefficient and gain of the simulated antenna are shown in Figs. 5 and 6, respectively. As shown in Fig. 5, an impedance mismatch was observed at some frequencies in the range of 4 to 8 GHz, for the antenna containing the single-unit cell. Therefore, for impedance matching, the antenna has been simulated with two-, three-, and four-unit cells (see Fig. 4b–d, respectively). The impedance matching, as shown in Fig. 4b, was improved, and we reached a suitable bandwidth in the frequency range of 4.2 to 7.5 GHz, and at a length of l2 = 82.4 mm. As seen in Fig. 5, the best impedance matching was also achieved with three-unit cells (Fig. 4c), with a length of l2 = 85 mm in the frequency range of 4.1 to 8.1 GHz, which indicates good impedance matching in this range, and also a wide bandwidth of 65%. Finally, the performance of the antenna has been investigated with four-unit cells, and it showed an impedance mismatch in the frequency range of 4–8 GHz. Figure 6 shows the gains for the four designed structures, which are slightly different due to their differences in dimensions.

**Figure 7:**
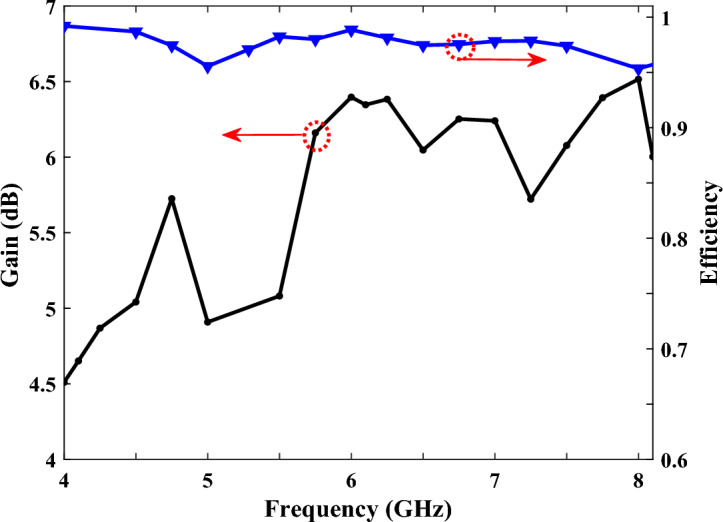
The simulated gain and efficiency of the SSPP antenna.

The results of the gain and efficiency of our proposed antenna are shown in Fig. 7. As seen in the figure, the total gain varies from 4.5 to 6.5 dBi, and the average efficiency is observed to be around 97%, over the whole operating band from 4.1 GHz to 8.1 GHz.

**Figure 8:**
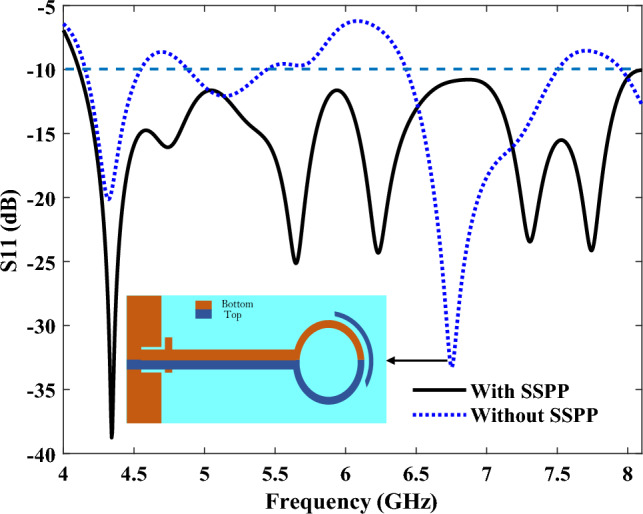
The reflection coefficient (S11) both with and without an SSPP waveguide.

It is worth highlighting that the use of SSPP transmission lines has been considered, here, due to their lower losses and impedance matchings. To better understand this, the results of the reflection coefficients of the structure, both with and without the SSPP, are shown in Fig. 8. By using the gaps at the beginning of the transmission line, which cause slow waves, and also by using SSPP, a series of new resonant frequencies is created, which by combining these frequencies with the resonant frequencies created without the SSPP transmission line, it has increased the bandwidth of the antenna. Moreover, the structure with the SSPP has a better performance due to having fewer losses, and the reflections are also suppressed below − 10 dB, within a wide frequency band from 4.1 to 8.1 GHz.

**Figure 9:**
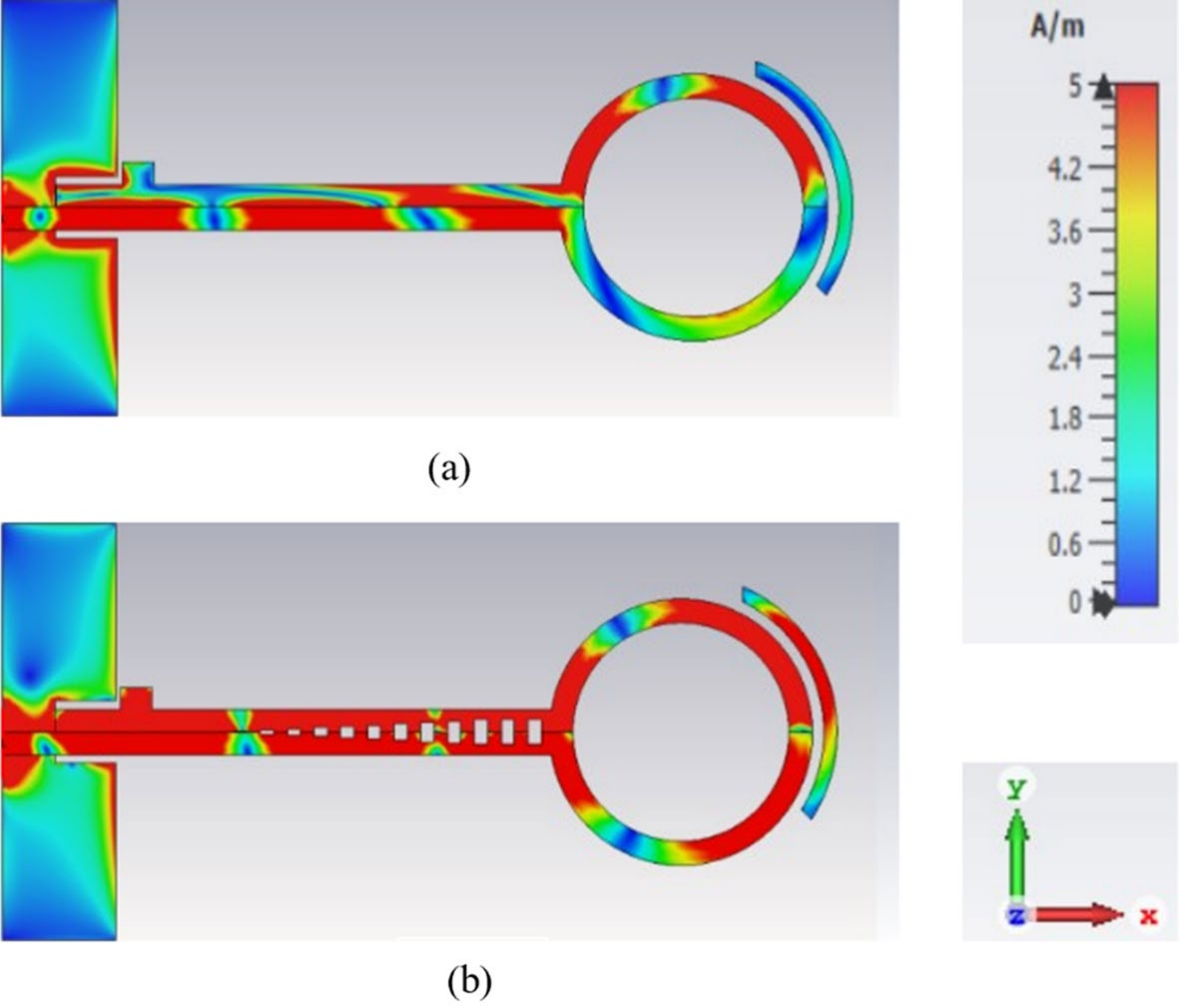
Electric field distribution at 6 GHz (**a**) without SSPP (**b**) with SSPP.

The distribution of the electric field for the frequency of 6 GHz with SSPP and without SSPP is plotted in Fig. 9. As can be seen, the field in the SSPP transmission line has more confinement, which has led to less loss in the antenna and ultimately caused better antenna radiation.

## Fabrication and measurement

**Figure 10:**
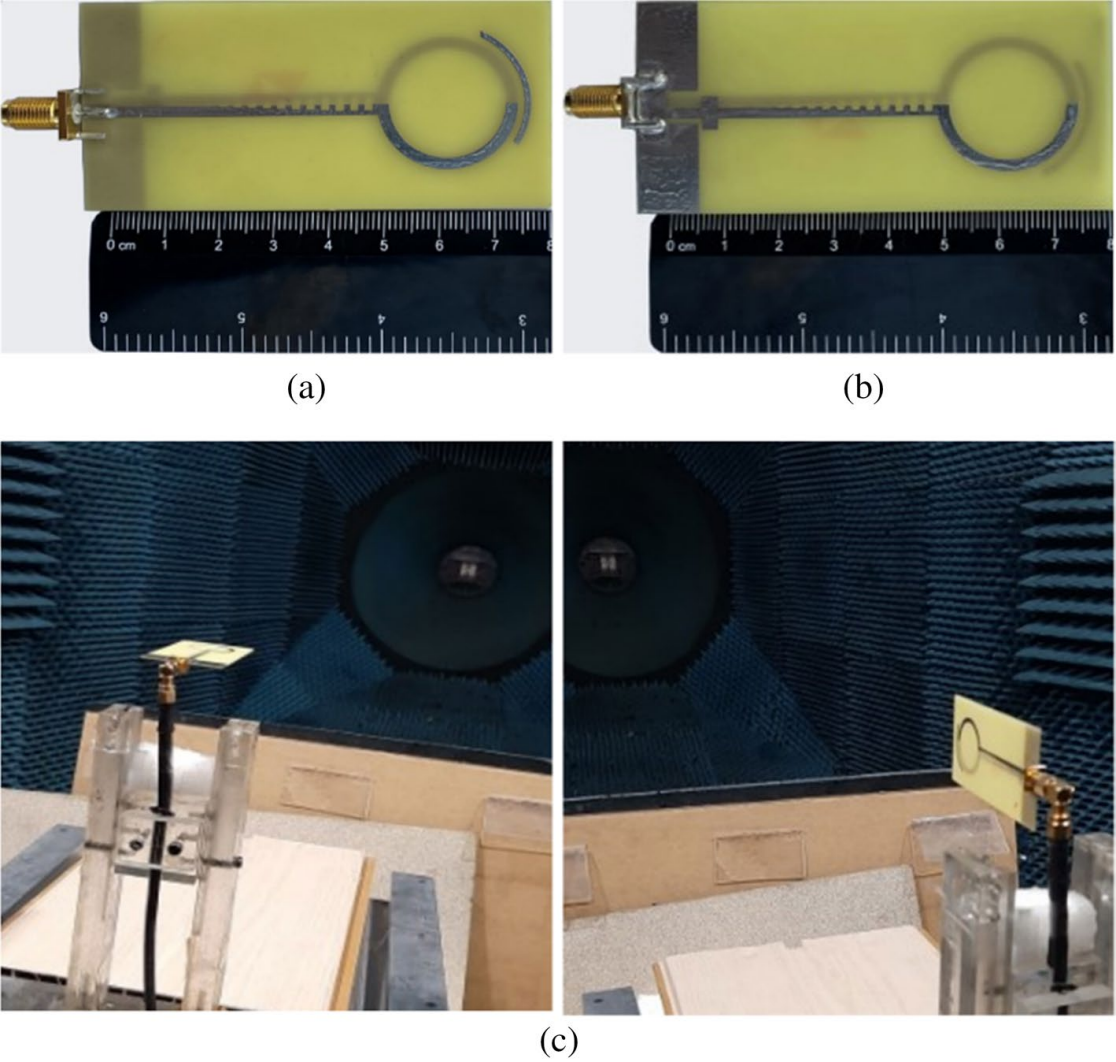
The prototype of the fabricated antenna. (**a**) Top view. (**b**) Bottom view. (**c**) A view of the antenna in the antenna room.

To experimentally verify the proposed wideband end-fire antenna based on an SSPP, as shown in Fig. 10, we have fabricated a prototype. As mentioned previously, the proposed antenna has been designed on a low-loss Rogers Ro4350B substrate. However, due to the unavailability of this substrate, the manufacturing and measuring have been performed on an FR-4 substrate, with a thickness of 1 mm, ${\varepsilon }_{r}=4.3$ and a loss tangent of 0.025. The other parameters were the same as in Fig. 1.

**Figure 11:**
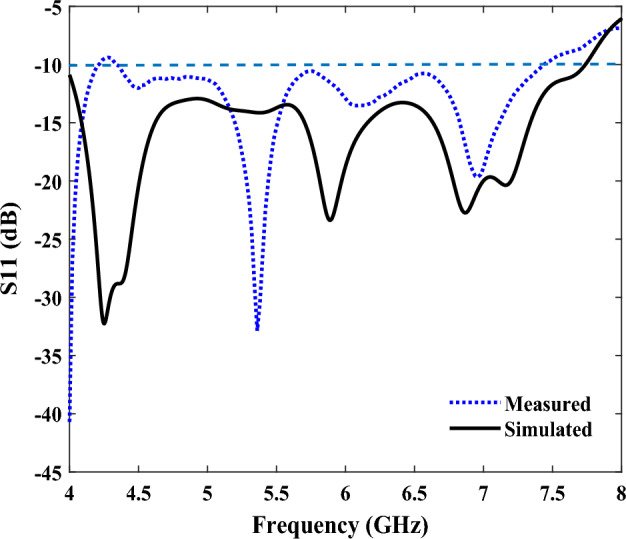
The measured and simulated reflection coefficients (S11) of the SSPP antenna.

**Figure 12:**
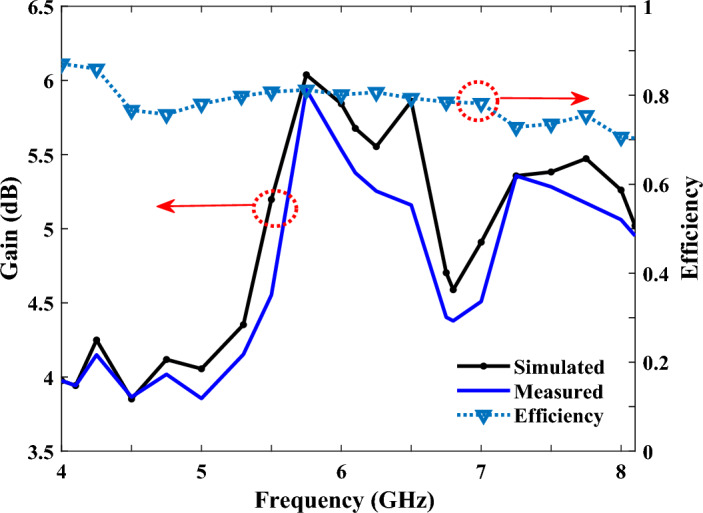
Simulated and measured gain results of the SSPP antenna, and calculated efficiency of the proposed structure.

**Figure 13:**
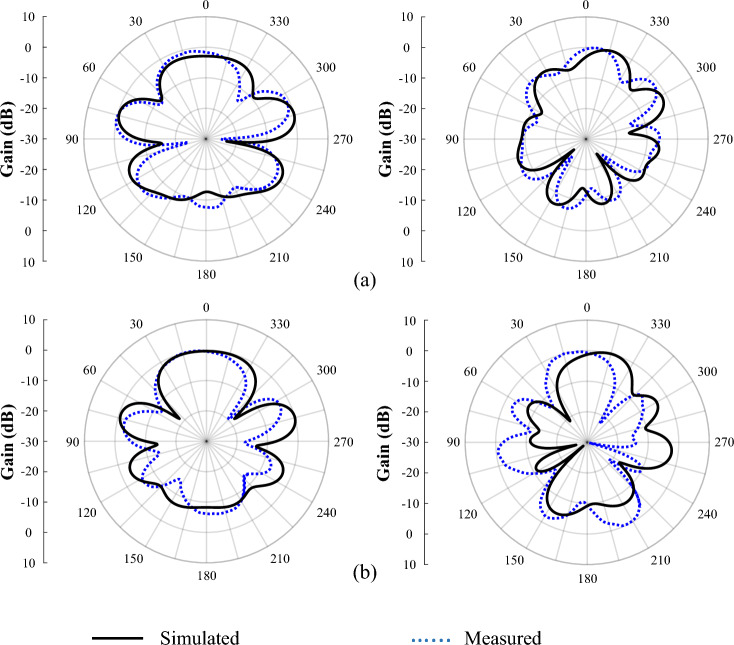
The simulated and measured normalized E-plane and H-plane radiation patterns, at different frequencies, for the proposed antenna [(right) E-plane; (left) H-plane]. (**a**) 6 GHz. (**b**) 6.8 GHz.

Figure 11 shows the simulated and measured reflection coefficients, which were consistent with each other, and the similarity of these results proves the design of our proposed antenna. Any resulting frequency deviations could have been caused by manufacturing errors. The reflection coefficient, in the range of 4 to 7.7 GHz, was below − 10 dB, representing a good impedance matching. Figure 12 also shows the simulated and measured antenna’s gains and efficiencies, in the frequency range of 4 to 8.1 GHz; and as can be seen, the results obtained from the simulation and the measurement have agreed well with each other. Importantly, as the FR-4 substrate has more losses compared to the Rogers Ro4350B substrate, the efficiency of the antenna on the FR-4 substrate has been reduced. Finally, the normalized, simulated and measured E-plane and H-plane radiation patterns, at the frequencies of 6 and 6.8 GHz, are illustrated in Fig. 13, which shows that they were in good agreement. Furthermore, the FBR parameter of the proposed antenna, which indicates the unidirectional radiation capability of the antenna, is shown in Table 3, and as seen in this Table, the simulated and measured results of the FBR for the proposed SSPP-based quasi-Yagi antenna have agreed well.

**Table 3:** The simulated and measured FBRs of the proposed antenna.

Frequency (GHz)	Simulated (dB)	Measured (dB)
6	10.5	10
6.8	10	10

In this paper, a simple structure based on SSPPs, with a wide bandwidth, and a high efficiency and gain has been presented. Since the electromagnetic waves are strongly confined around the metal surface of this structure, the proposed antenna has low loss. Table 4 depicts the comparison of our proposed antenna with other works in the literature.

**Table 4:** A comparative analysis with other sources.

References	Bandwidth (%)	Efficiency (%)	Gain (dBi)	Frequency (GHz)	Size (mm)
^7^	43*.*3	92	6*.*43	6	50 × 40
^12^	55	96	9.2	8	107 × 30
^21^	8.5	92.32	7	6	113 × 32
^23^	32.1	95	9.16	7	133 × 32
This work	65	97	6.5	6	85 × 40

## Conclusion

In this paper, a compact quasi-Yagi antenna, based on an SSPP, has been designed, simulated, and fabricated. An SSPP waveguide has been used due to their ability to provide field confinement, slow waves, and low losses for the transmission line, and to obtain wide bandwidths. A high efficiency of 97%, with a wide bandwidth of 65%, and a peak gain of 6.5 dB has been obtained with the proposed antenna simulated on a Rogers Ro4350B substrate. However, due to the unavailability of this antenna has been performed on an FR-4 substrate. The bandwidth, maximum gain, and efficiency obtained from the simulation and measurement with the FR-4 substrate were approximately 61%, 6.1 dB, and 77%, respectively. Thus, based on the advantages of the SSPP transmission line, our proposed antenna with a wide bandwidth, high efficiency, and compact size, shows great potential for use in wireless communications.

## Data Availability

The datasets generated and analyzed during the current study are available from the corresponding author on reasonable request.
